# Monthly Summary

**Published:** 1885-08

**Authors:** 


### MONTHLY SUMMARY.

GALVANISM FOR NEURALGIA.—Dr. Mattison, of Brooklyn, calls attention to the value of galvanism for the relief of neuralgic pain, not because there is anything new in the treatment, but to point out the fact that electricity may often be used instead of morphia, and spare the patient the danger of contracting the opium habit. He has found that very weak currents only are required in most cases, and laments the absence of small galvanic batteries easy to carry about, believing that the bother of transporting the galvanic batteries of the usual "portable" size often prevents physicians from trying the remedy.

The method of application which he recommends is to place the positive electrode over the point of issue of the affected nerve from the skull or spinal column, and the negative pole over the seat of pain. About the head and face the strength of the current should be such, as, when broken, to cause a metallic taste in the mouth, or slight flashes of light. The usual length of sitting is between five and fifteen minutes, but sittings shorter than five minutes may be sufficient for the relief of the pain, or it may be advisable to continue a sitting beyond fifteen minutes if there be steady relief of the pain under the application. The frequency of the sitting should depend upon the frequency of the attack, every paroxysm of pain being met with an application of electricity when possible. As an illustration, a case is quoted from Dr. Tibbitts where a patient had suffered from neuralgia for two years having from six to twenty attacks daily. On the first day the electricity was applied twenty times. Improvement was rapid; the applications were reduced in number in correspondence with the diminished frequency of the attacks, and at the end of a month were made but twice a week. In three months the patient was cured.—*Iowa State Med. Reporter.*

---

EFFECT ON OFFSPRING OF CONSANGUINEOUS MARRIAGES.—Dr. Charles F. Withington, of Boston, presented at the recent meeting of the Massachusetts Medical Society a tabulated report of 108 consanguineous marriages. 413 children were born from 103 couples. Considering as "unhealthy" even those having strabismus, those developing phthisis late in life, those who were below the average in intelligence or bodily vigor and those who died in infancy, there remained 312 healthy children. There were 12 cases of deaf-mutism, 7 of insanity, 13 of idiocy and 15 of phthisis. In 17 cases one or both of the parents were themselves descended from a consanguineous union. 15 were fertile, producing 68 children, of whom 48 were healthy. The view was announced that the evil results were due to the operation of the ordinary laws of morbid inheritance, and that consanguinity *ipso facto* had no influence either for good or evil.—*N. Y. Med. Journal.*

